# Long-term apical bone gain after implant placement combined with internal sinus-floor elevation without graft

**DOI:** 10.1186/s12903-020-01178-4

**Published:** 2020-07-09

**Authors:** Peter Rammelsberg, Julia Pahle, Christopher Büsch, Andreas Zenthöfer

**Affiliations:** 1 Dental School, Department of Prosthodontics, University of Heidelberg, Im Neuenheimer Feld 400, 69120 Heidelberg, Germany; 2 Zahnarztpraxis Dr. Schröder & Partner, Kronenstrasse 20, 70173 Stuttgart, Germany; 3 Dental School, Department of Prosthodontics, University of Heidelberg, Im Neuenheimer Feld 130.3, 69120 Heidelberg, Germany

**Keywords:** Implant, Sinus-floor elevation, Internal, Bone gain

## Abstract

**Background:**

To assess changes in apical bone height/bone gain over up to 8 years after implant placement combined with simultaneous internal sinus-floor elevation (ISFE) without use of graft.

**Methods:**

217 implants were placed in combination with graft-free ISFE and successfully healed in the posterior maxilla of 138 patients. Radiographs after surgery across an evaluation time of up to 8 years were analyzed. Changes in apical and marginal bone height related to the implants were measured. Differences in bone height over the study period were evaluated by use of Wilcoxon tests. To identify possible influencing factors on apical bone gain and marginal bone loss, backward linear regression variable selections and linear mixed regression models were performed.

**Results:**

At the apical aspects of the implants, significant mean vertical bone gain of 2.4 mm (mesial) and 2.6 mm (distal) was observed after 6 months (*p* < 0.05). Radiographic analysis yielded additional bone gain of up to approximately 3.5 mm over the study period. Small initial bone height was crossed with more pronounced apical bone gain (*p* < 0.05).

**Conclusions:**

If implants are placed combined with graft-free ISFE, significant vertical bone gain, especially in the first year post-op, can be expected. Smaller initial bone height is associated with a higher likelihood for greater bone gain.

## Background

Dental implants are nowadays routinely used to support single crowns, fixed or removable dental prostheses. In the posterior maxilla, treatment by use of endosseous dental implants is frequently compromised due to inadequate bone volume because of a large volume maxillary sinus. Boyne et al. [1] and Tatum [2] described a technique for sinus floor elevation including a lateral window access to the sinus floor and manipulation of graft material below the Schneiderian membrane in order to increase bone volume. In the case residual bone volume would allow primary stability of a implant, simultaneous implant placement was recommended [3].

The internal / transcrestal sinus-floor elevation (ISFE), introduced by Summers [4], is a less invasive approach. Instead of the lateral window, sinus floor is accessed through the drill hole generated by use of a pilot drill. Afterwards the Schneiderian membrane is elevated by use of hand osteotomes pushing graft material up. The Summers technique has been recommended as a predictable alternative for implant placement in maxillary bone of at least 6 mm in height [5]. In an early review, Tan et al. [6] estimated 3-year survival of 92.8% for implants placed in combination with ISFE. Shrinkage and a re-modelling processes linked to loss of graft height were detected for augmentations using grafts during the first 1 to 3 years post-op [7–9]. In 2010 a systematic review on the effectiveness of different sinus-lift approaches complained about limited and underpowered clinical studies [10].

The need for use of graft material for sinus-floor elevation has been taken into question. New bone formation at the apical aspects of the implants was observed in the absence of graft material [11, 12]. A recent systematic review taking closer look at implant survival after lateral sinus-floor elevation after 48 to 60 months was similar irrespective of graft (99.6%) or graft-free (96%) approach [13]. For the transcrestal / internal approach without graft, a previous study suggested that a gain of apical bone height of 2 and 3 mm can be expected after 6 months and 3 years, respectively, with no significant difference in implant success compared to use of graft material [14]. Another study observed similar mean apical bone gain after 10 years of clinical observation. The greatest bone gain was seen within the first year after surgery [15]. However, sample sizes of both studies were low (< 23 implants analyzed). A further study with larger sample sizes found mean bone gain of up to 2 mm after ISFE without graft [16], again similar to other thematic studies. However, here the observation period was rather short with 17-months follow-up. With regard to implant prognosis, the authors of this study also reported that residual bone heights lower than 4 mm and membrane perforations reduce the probability of 10-year survival [17].

In meanwhile, a systematic review dealing with graft-free sinus-floor elevation included 22 studies (*n* = 864 implants) with a lateral or transcrestal approach [18]. Mean implant survival was 97.9% and residual bone height was in mean height approximately 6 mm. Weighted mean vertical bone gain was 3.8 mm and mean marginal bone loss around the implants was 0.91 mm. Significantly more bone gain was observed for long implants and implants placed combined with a lateral access window [18].

Many studies included limited sample sizes combined with short-term observation periods, predominantly several months after prosthetic restoration [19]. This shortcoming was also addressed in a recent review [20]. Shrinkage of newly formed bone after ISFE without graft has rarely been addressed. Furthermore, long-term evaluations of apical bone gain are rare.

The objective of this observational cohort study was, therefore, to evaluate longitudinal changes in bone height, apical bone gain, and marginal bone loss around implants placed combined with graft-free ISFE by evaluation of radiographs. Predictive factors for apical bone gain and marginal bone loss were also determined. Based on preliminary research [14–16] it was hypothesized that bone gain at the apical aspects of implants can be expected after internal sinus-floor elevation without graft after the healing period as well as in the long-term.

## Methods

The study protocol was evaluated and approved by the local ethics committee of the University of Heidelberg (registration number: 229/2005).

### Study population

Between April 2003 and December 2009, patients received a total of 225 implants in combination with simultaneous graft-free ISFE at the Department of Prosthodontics, University of Heidelberg. Patients with contraindications against implant surgery (i. e. ongoing radio-chemotherapy, active sinusitis) were not served with implants. The participants’ inclusion criteria for the clarification of the present research question were use of successfully healed Straumann (Waldenburg, Switzerland) tissue-level implants, and consecutive radiographs from baseline and from at least three follow-ups. With respect to implant surgery, the minimum residual bone height to the sinus had to be 2 mm. No further exclusion criteria were stated, smokers were considered in the study, too.

The length of the implants was 8 mm for 12 implants, 10 mm for 187 implants, and 12 mm for 18 implants. The diameter was either 4.1 mm (*n* = 133) or 4.8 mm (*n* = 84). The surgeon selected the dimensions of the implants to be placed according to vertical and horizontal bone volume as clinically and radiographically estimated. One hundred and twenty-seven implants were placed in the molar region and 87 in the premolar region. Only five implants were placed further anteriorly, when the sinus was extended deeply into the canine region.

### Surgical procedures

The surgical procedures are also described in previous works [16, 17]. After local anesthesia, mid-crestal incision was performed and buccal / palatal full-thickness flaps were reflected. A surgical splint was used to mark implant position with a round bur (1.4 mm in diameter), and a pilot drill (2.2 mm in diameter) was used to define the angle of the implant. The pilot drill ended approximately 1 mm below the sinus floor, as calculated from the pre-surgical x-ray (by use of length-corrected measuring distances in the imaging software). No additional intra-operative x-ray control was performed with a drill or a depth gauge in situ. Preparation of the recipient beds was conducted by use of subsequent spiral drills (according to the diameter of the planned implant 2.8, 3.5 to 4.2 mm, e.g. last drill for 4.1 mm diameter implant at maximum 3.5 mm). Finally, a parallel hand osteotome was used under gentle malleting force to cause initial fracture of the sinus floor. The osteotomes had no length stoppers but a millimeter scale with a bold mark in the region of 10 to 12 mm. The sinus floor was then elevated by use of the hand osteotome (diameter 2.8 mm), displacing the Schneiderian membrane apically. This step was performed manually with special attention paid to avoid perforation of the membrane. Depending on the extent of the sinus floor elevation and bone quality the recipient site was extended by use of the consecutive hand osteotomes and / or spiral drills. All implant insertions were performed by use of a hand ratchet. Minimal insertion torque of 5 Ncm was mandatory for implant placement and consideration in this study. Even if perforation of the Schneiderian membrane was detected, the entire implant insertion procedure was conducted without further treatment. The integrity of the Schneiderian membrane was assumed if the depth gauge with 2.8 mm in diameter was elastic in the drill hole and vice versa. In case of doubt possible perforations were re-checked using nasal patency test and by a look at the post-op x-ray. All implants were covered with respective healing screws at tissue level (not submerged). Flaps were repositioned and closed by use of single button sutures (edentulous spaces) or vertical mattress sutures in sites between two implants or if implants were neighbored by natural teeth (creation of papillae-like soft tissue) [21].

As prophylaxis, all patients received 3 × 1000 mg Amoxicillin for 6–7 days and analgesics as required. Oral hygiene was performed as normal, except for tooth-brushing around the implants for 7 days. Sutures were removed 6–9 days after surgery. Implants were placed by four experienced dentists or under supervision of one of the experienced dentists. After an unloaded healing period between six and 12 months (according to the extent of sinus-floor elevation and bone quality), all implants were restored with single crowns, or with fixed or removable dental prostheses. Albeit the Schneiderian membrane is osteogenic, for sinus floor elevation with e.g. 2 mm residual bone height to the sinus / small insertion torque (e.g. 5 Ncm) a prolonged waiting time until the incorporation of the final prostheses was scheduled to allow bone adequate healing.

### Radiographic evaluation

All patients underwent panoramic radiographic examination before surgery. Immediately after implant placement again radiographs were conducted. All panoramic and single radiographs were taken by use of digital detectors. Single radiographs were standardized by use of parallel technique and respective film-holders. Sidexis software (Sirona, Bensheim, Germany) was used to measure and calculate correction factors. One dentist who was not involved in implant placement and prosthetic restoration evaluated the radiographs taken at baseline (post-op, T0) and at the follow-up visits after six (T1) and 12 months (T2) as well as after two (T3), three (T4), four (T4) and 5 to 8 years (T6) following implant surgery for the presence or absence of bone gain or loss at the apical and coronal aspects of the implants (in mm). To assess reliability, repeated measurements were performed for 40 implants by a second investigator unaware of the initial results. In the majority of cases post-op radiographs were panoramic in nature whilst single intraoral radiographs were often taken at the follow-up visits.

**Fig. 1:**
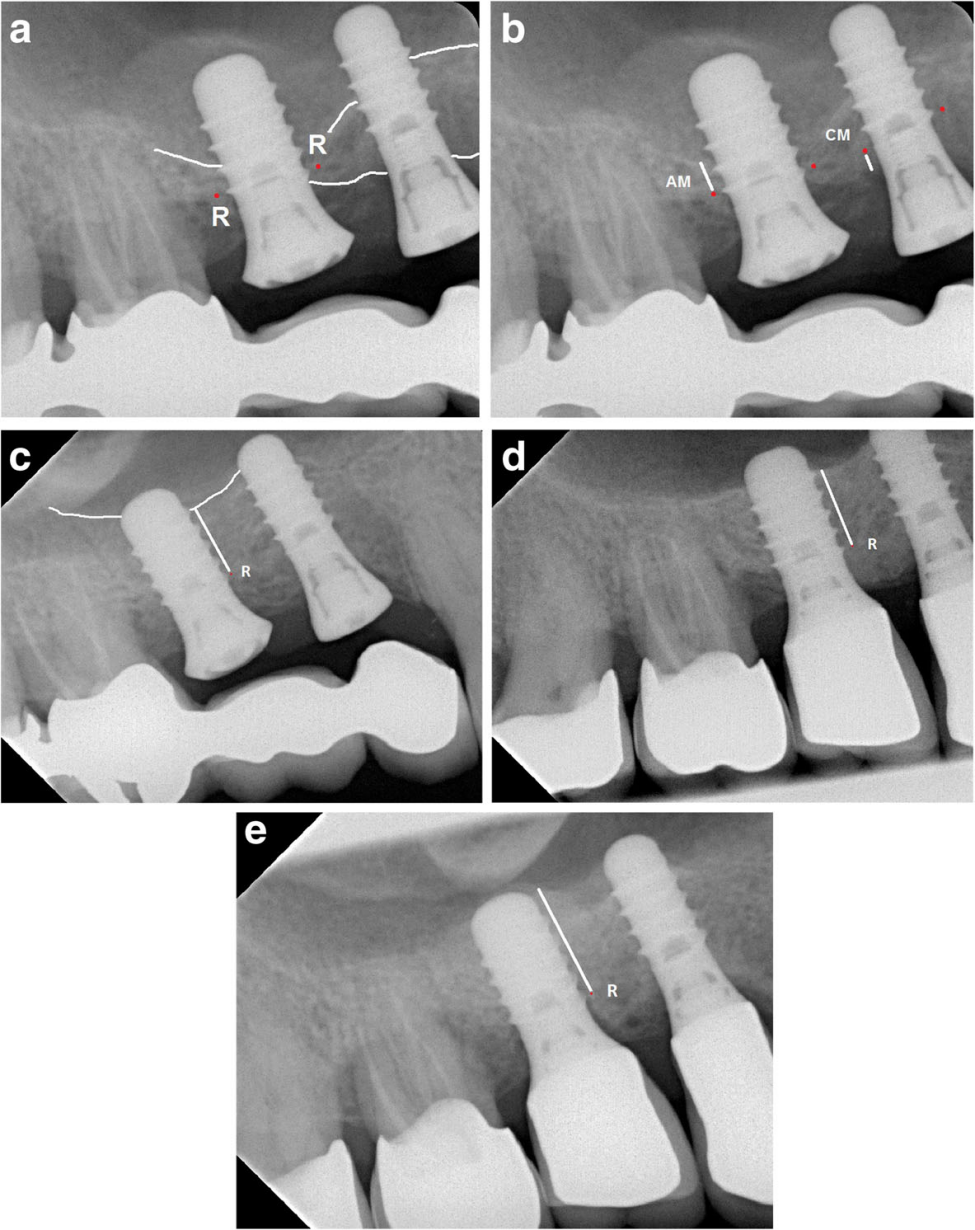
Radiographs showing measurements of bone heights (bone edges, marginal and sinus floor, with white lines) using the first coronal thread as reference point R (red dots). **a** post-operative; **b** after 6 months; **c** after 2 years; **d** after 6 years; **e** after 10 years. Legend: R = reference point (first coronal thread of the implant); AM = apical measurement (distance between R and the sinus floor); CM = coronal measurement (distance between R and the marginal bone edge). Note: For reason of clarity R, AM and CM are sketched at two implants. The complete measurements (mesial and distal) were performed for all implants. In the radiograph **c**) it can be seen that the implants are still not loaded. In some cases incorporation of final prostheses was prolonged due to participants’ causes, e.g. financial restrictions

The first (coronal) thread of the implant was used as reference point (R) to measure apical bone height at the mesial and distal aspects of the implants (AM). Because the real length of the implant was recorded, measurements were corrected by the individually determined enlargement factor of the radiograph. Apical bone height was measured from R apically to the bony sinus floor, at the mesial and distal sides of the implant. See Fig. 1a+b for details. Apical bone gain was assessed by calculating changes in bone height from baseline (post-op, T0) to the follow-ups from T1 to T6 using the corrected differences between the absolute values at different times. Radiographs at T1 were acquired before prosthetic restoration, and subsequent radiographs were acquired after prosthetic restoration (Figs. 1a-e). Compare also [16, 17] for radiographic measurement procedures.

Marginal bone loss was measured (CM) simultaneously, again using R as reference. The distance from R to the marginal bone level was calculated from T0 to T6 by using the correction factor as described for the apical measurements. See Figs. 1a-e.

### Statistical analysis

Statistical analysis was done with the aid of SPSS (Version 22; IBM, IL, USA) and R (Version 3.6.1; R Foundation, Toulouse, France). Descriptive data was reported according to structure and distribution of target variables as numbers (%) or means (SD). Additionally, selected target variables were pictured by use of box plots. Inter-examiner reliability was assessed on the basis of 40 implants with repeated measurements by two investigators using Pearson correlation analysis (compare [16]). Differences between bone heights at different evaluation appointments (baseline / follow-ups) were compared by use of Wilcoxon tests for matched pairs. Linear mixed regression models with random effects were compiled to evaluate possible influencing variables on changes of apical (1) and coronal (2) bone heights along the study visits. The models (1) and (2) were each run separately for the mesial and the distal aspect, respectively, of the implants. The implant number per patient and the patient itself were included in the models as random effects to care for a possible bias due to unequal numbers of studied implants per participants or a possible influence of specific implant characteristics (e.g. width). A backwise selection of the possible independent influencing variables was performed using the ‘Akaike information criterion’. The full model each was: Y = age + sex + implant length + measurement time + baseline bone height. *P*-values smaller than p of 0.05 were regarded as statistically significant.

## Results

Of 225 tissue-level implants initially placed in combination with simultaneous internal sinus-floor elevation without graft, 217 implants were considered for the radiographic analysis. The eight excluded implants could not be considered for statistical analyses (no series measurement of radiographic changes in bone height possible) due to early failures (incidence of failure, absence of bony healing: 3.6%).

**Fig. 2:**
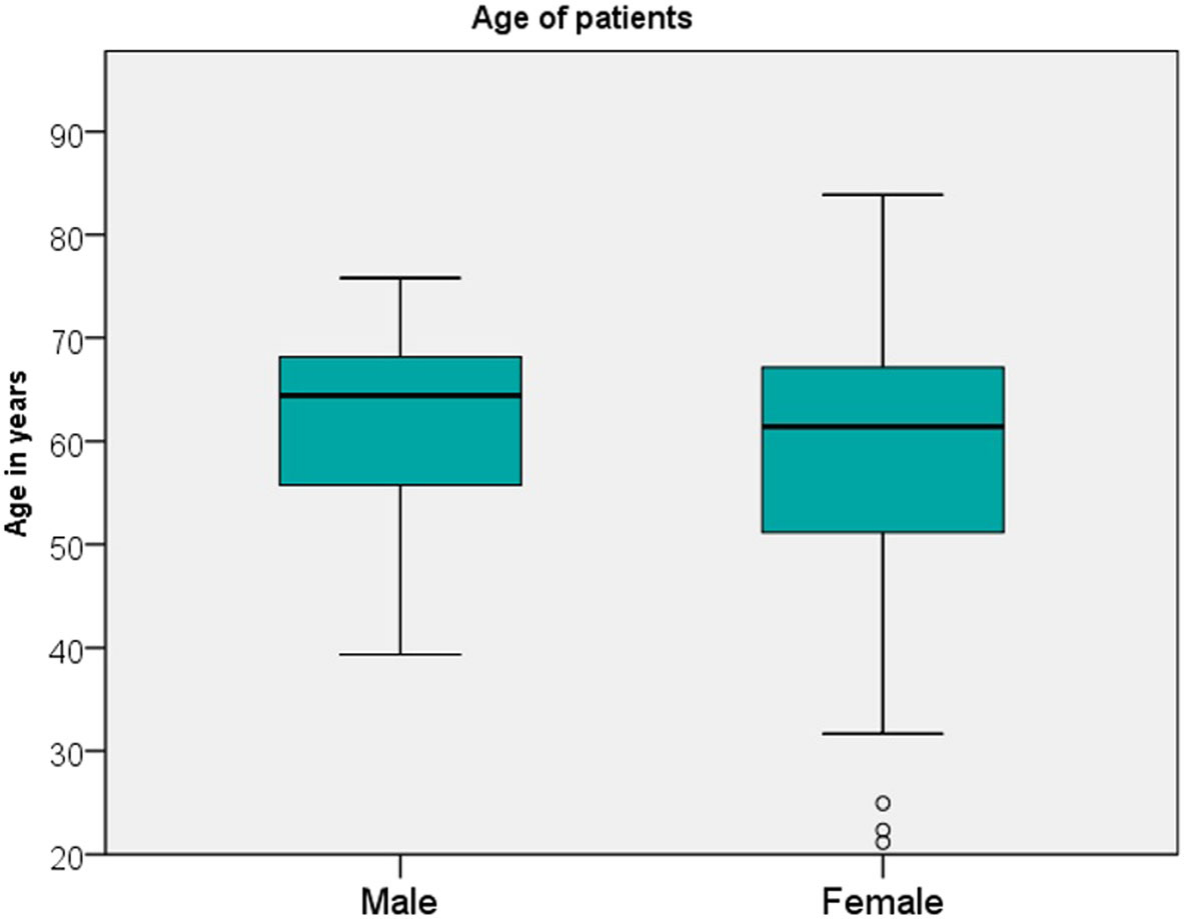
Variation in age of female and male participants

Thus, the study population consisted of 67 (48.6%) male and 71 (51.4%) female patients with a mean age of 60.0 years (minimum: 21.2 years; maximum: 83.9 years) from a prospectively documented clinical study. See Fig. 2 for the variation of age in male and female participants. Mean (SD) healing time / waiting time until incorporation of prostheses was 9.2 (4.3) months. At baseline some 7% of the participants were smokers.

Apical and coronal bone height of 40 implants was measured by two independent investigators. High correlation coefficients ranging from 0.808 (coronal bone height at the mesial aspect) to 0.897 (apical bone height at the mesial aspect) indicated good inter-rater reliability of bone height measurements at the mesial and distal aspects of the implants.

Radiographs from at least three follow-ups ranging between 6 months and 8 years were available for 217 implants. Follow-up intervals ranged from 6 months (T1) to 4 years (T5); T6 included all implants with follow-up radiographs from 5 to 8 years after surgery. Because radiographic evaluation was not performed at every follow-up, the sample size of implants with radiographs ranged from 57 to 148.

Mean total bone height at baseline was 6.8 mm and 6.1 mm, respectively, at the mesial and distal aspects of the implants. In the few cases where bone height was above 10 mm at the mesial side, ISFE was used for the distal aspect of the implant only (range 1.5–12 mm).

**Fig. 3:**
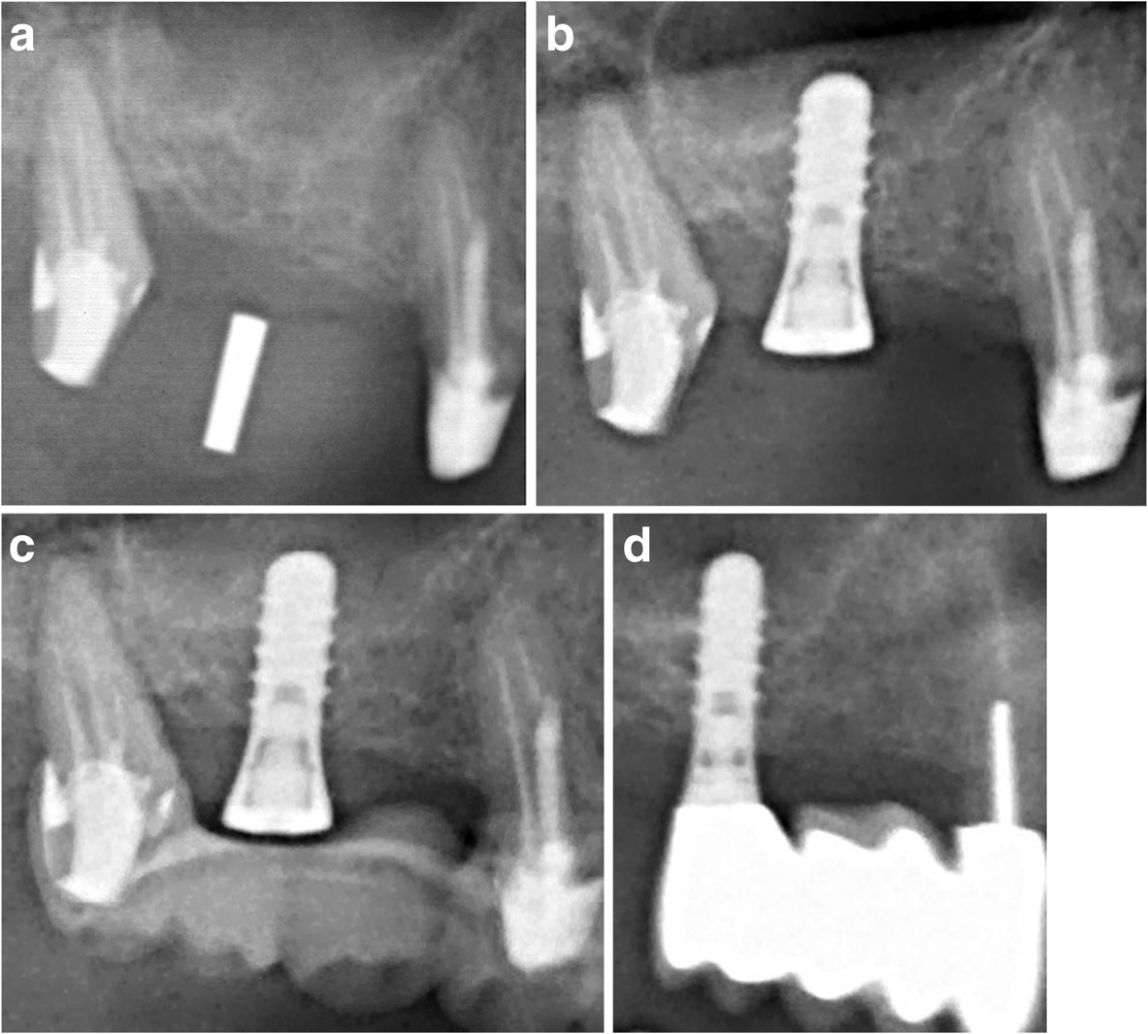
**a**-**d** Radiographs showing an exemplary participant case from the study, in **a** the radiologic planning is shown, **b** shows the radiograph after implant surgery, **c** shows the radiograph after 6 months and **d** after 10 years in clinical service

An exemplary patient case for visualization of the development of bone heights is shown in Fig. 3.

**Fig. 4:**
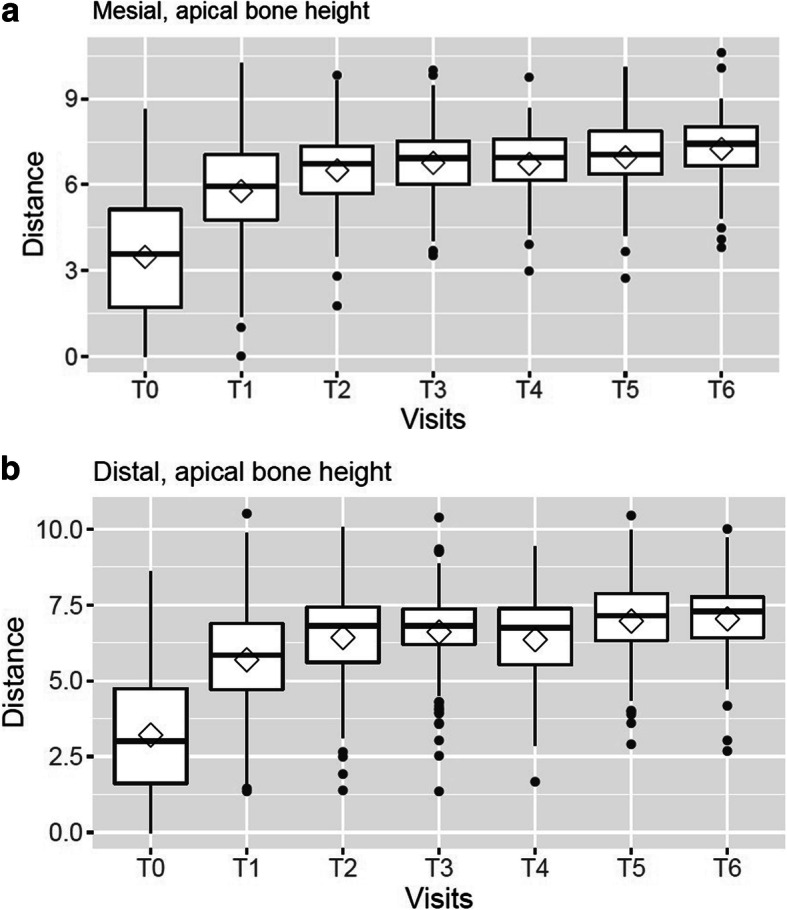
**a**+**b**. Variation in apical bone height (mm) from baseline (T0) to T6 (end of study). Measured from the first thread of the implants at the mesial (**a**) and distal (**b)** aspects of the implants

From baseline (T0) to T6, mean apical bone height measured from the first thread of the implants increased from 3.5 mm to 7.3 mm at the mesial side and from 3.2 mm to 7.0 mm at the distal aspect. Fig. 4 shows the changes in bone height between the implants from T0 to T6. Statistical comparison of apical bone height from baseline and follow-ups by use of paired Wilcoxon tests revealed significant differences between baseline and all follow-up measurements (*p* < 0.001). Furthermore, significantly higher values were recorded for T2 than for T1 (mesial: *p* = 0.001; distal: *p* = 0.002). Taking *p* < 0.01 as the level of significance, because of multiple testing between groups, a further significant increase in bone height was only found between T3 and T4 for the distal side of the implants.

Vertical bone gain was calculated on the basis of individual differences between apical bone height at baseline and at each follow-up. Vertical bone gain increased from 2.44 mm at T1 to 3.63 mm at T6 at the mesial aspects of the implants, and from 2.62 mm to 3.48 mm at T5 at the distal aspects After T4, only minimal variation in mean apical bone gain was observed (Table 1).

**Table 1:** Mean and standard deviation (SD) of apical bone gain (in mm) from T1 to T6

Location	Time	*n*	Mean	SD
Mesial	T1	148	2.44	2.16
	T2	101	2.75	2.06
	T3	93	3.03	1.87
	T4	74	3.44	2.26
	T5	56	3.38	2.25
	T6	66	3.63	2.24
Distal	T1	148	2.62	2.06
	T2	101	2.94	2.12
	T3	94	3.17	1.99
	T4	74	3.44	2.31
	T5	56	3.48	2.30
	T6	66	3.34	2.01

Perforation of the Schneiderian membrane occurred during preparation of 47 out of 217 implants (21.7%). Because elevation of the sinus floor was successful in these cases, implants were placed in accordance with the regular protocol. Apical bone gain was also calculated separately for implants with perforated and non-perforated membranes. A similar pattern of distribution of values for apical bone gain was observed for both groups. Over the study period (T0-T6) a difference of bone gain in favor of implants placed with non-perforated membranes compared to perforated membranes was 1.2 (mesial) and 0.6 (distal) mm. These differences were not statistically significant (*p* > 0.08).

The mixed model for changes of apical bone height of the mesial aspect of the implants yielded that bone gain was significantly affected by less initial bone height at baseline (*p* = 0.0001). The study visits T0-T6 also had a significant effect on changes in bone height, with an increase in height over time (*p* = 0.0001). Implants of 10 and 12 mm in length were crossed with greater bone gain compared to 8 mm long ones (*p* ≤ 0.0001). No other possible influencing factors reached the level of statistical significance (*p* > 0.05). The analog model for the distal aspect of the implants drew the same picture. Less baseline bone height, greater implant length and time of follow-up visit had a significant effect on greater bone gain (*p* < 0.0001) whilst the other variables had no effect. See Table 2 for detailed results.

**Table 2:** Linear mixed model for apical bone changes as dependent variable and possible independent influencing variables and ‘patient’ and ‘implant number’ as random effects (the end model after selection process is shown)

Variable	Estimate	95% CI LB	95% CI UB	*P* value
Mesial				
Visit time (T0-T6)	0.2558	0.2068	0.3047	0.0000
Baseline bone height	−0.7880	− 0.8583	− 0.7178	0.0000
Implant length 10 mm	1.3866	0.7024	2.0707	0.0001
Implant length 12 mm	2.4587	1.6105	3.3069	0.0000
Distal				
Visit time (TO-T6)	0.2366	0.1868	0.2864	0.0000
Baseline Bone height	−0.7637	−0.8535	− 0.6739	0.0000
Implant length 10 mm	1.6216	0.8308	2.4123	0.0001
Implant length 12 mm	2.8623	0.1.885	3.8360	0.0000

Legend: *T* visit time, *CI* confidence interval, *LB* lower border, *UB* upper border

The non-significant variables were removed from the table in terms of clarity

Measurements of coronal bone height from the first thread to the marginal bone level were used to calculate longitudinal marginal bone loss. Coronal bone height decreased significantly (*p* < 0.001) from T0 compared to all follow-ups. Later reductions of coronal bone height between follow-ups were not significant, with *p*-values ranging between 0.091 and 0.895).

Marginal bone loss calculated on the basis of individual differences between marginal bone height at follow-up and baseline resulted in mean bone loss of − 0.86 mm mesial and − 0.80 mm distal at T1. Further mean marginal bone loss was minimal; the highest value calculated was − 1.41 mm after 6–8 years at T6 (Table 3).

**Table 3:** Mean and standard deviation of marginal bone loss (mm) calculated on the basis of individual differences in marginal bone height between follow-ups and baseline (T0)

Location	Time	*n*	Mean	SD
Mesial	T1	148	−0.86	1.10
	T2	101	−0.81	0.92
	T3	94	−0.99	1.15
	T4	74	−1.19	1.30
	T5	57	−0.97	1.31
	T6	66	−1.41	1.33
Distal	T1	148	−0.80	1.06
	T2	101	−0.84	1.02
	T3	94	−0.84	0.99
	T4	74	−0.97	1.18
	T5	57	−0.72	0.97
	T6	66	−1.19	1.00

The mixed model for changes of coronal bone height of the mesial and the distal aspects of the implants showed that less initial marginal bone height was crossed with less bone loss over the study period (*p* ≤ 0.0001). Manageable but significantly greater bone loss was linked to later follow-up visits (*p* < 0.0063). Other variables were not statistically significant (See Table 4).

**Table 4:** Linear mixed model for marginal bone changes as dependent variable and possible independent influencing variables and ‘patient’ and ‘implant number’ as random effects (the end model after selection process is shown)

Variable	Estimate	95% CI LB	95% CI UB	*P* value
Mesial				
Visit time (T0-T6)	−0.0661	−0.1005	−0.0317	0.0004
Baseline bone height	−0.6672	−0.7864	− 0.5480	0.0000
Distal				
Visit time (TO-T6)	−0.0451	−0.0774	− 0.0128	0.0063
Baseline bone height	−0.5436	−0.6757	− 0.4114	0.0000

Legend: *T* visit time, *CI* confidence interval, *LB* lower border, *UB* upper border

The non-significant variables were removed from the table in terms of clarity

Combining apical bone gain and marginal bone loss up to 8 years resulted in positive values for the complete observation period up to 8 years. At the mesial aspects, mean total bone gain increased from 1.60 to 1.97 mm between T1 and T2, and further increased up to 2.34 mm at T5. At the distal aspects, mean total bone gain increased from 1.81 mm at T1 to 2.70 mm at T5. After more than 4 years, mean total bone gain decreased slightly at both sides to 2.26 mm at the mesial aspects and to 2.15 mm at the distal aspects.

## Discussion

The research hypothesis of long-term bone gain following implant placement in combination with ISFE without graft must be accepted. The greatest bone gain can be especially expected within the first year of observation.

However, some aspects are requiring discussion. The implants selected for this analysis were placed, restored and prospectively documented in one center. Thus, variations in surgical and prosthetic procedures were reduced to a minimum compared to multi-center studies. Because selected implants were restricted to Straumann tissue-level implants with sandblasted large-grit and acid-etched surface, implant-related confounding effects were avoided. In a recent review [22], negative effects of smooth or machined surfaces on the implant prognosis have been shown. This factor and its possible confounding effect were eliminated in our study. On the other hand, however, representativeness of monocentric data is limited.

Substantial apical bone gain was observed for maxillary implants placed combined with ISFE without graft. In their review, Perez-Martinez et al. [23] calculated a mean apical bone gain of 3.4 mm after ISFE without graft material. In our study, a comparable value was calculated for apical bone gain after an observation period of 4 years. A different review [17], however, reported variation in vertical bone gain between 1.7 mm and 4.1 mm in non-graft groups after 3 years. On the basis of our study’s findings, variation in vertical bone gain was predominantly affected by the amount of residual bone height. A significant relationship in the regression analyses indicates that small residual bone height favors more bone gain. Because implant length was predominantly 10 mm, residual bone height was strongly related to the implant length protruding below the membrane. In a meta-analysis [18], the length of the implant reaching into the former sinus volume beneath the Schneiderian membrane was significantly correlated (Spearman *rho* = 0.8) with the amount of apical bone gain. The authors also calculated negative correlation coefficients for residual bone height and apical bone gain, but did not reach the level of significance. Greater variability in the implant length used in the different studies under review might explain this difference. With high variability in implant length, the strong mathematical relationship between small residual bone height and longer aspects of the implant protruding into the sinus volume was not observed.

If grafts were used for sinus-floor elevation, previous studies have described a remodeling process of the graft expressed by shrinkage and loss of height during the first 1 to 3 years after augmentation [7–9]. On the basis of cone-beam computer tomography and volumetric measurements, shrinkage of the grafted volume was confirmed in a prospective study by Markovic et al. [24] comparing different graft materials and a control group without graft. The authors observed substantial shrinkage of the endo-sinus bone 1 year and 2 years after implant placement, indicating significantly more shrinkage in grafted sites. The recent study revealed high initial vertical bone gain after 6 months post-op combined with decelerated but ongoing bone gain up to 4 years, if an ISFE technique without graft was used. Signs of long-term apical bone loss were not observed, apical bone height remained stable without signs of bone shrinkage in the long-term. This might be explained by fact that the transcrestal graft-free approach creates a new space underneath the Schneiderian membrane, preserved by the implant apex. This space fills up with blood; local native bone formation into this space is actuated (bone cells, growth factors). If grafts are used, autogenous and alloplastic / allogenic materials or a combination of both are conceivable. With autogenous grafts in advantage both approaches lead to favorable implant success [7–9]. Endochondral bone - which is frequently harvested for that purpose because of good availability - includes a prolonged revascularization time compared to membranous bone grafts. However, revascularization time plays a crucial role for resorption and therefore shrinkage of the graft [25]. In contrast, alloplastic / allogenic grafts rather represent a lead structure for bone formation than they are osseoinductive. Bovine hydroxyapatite for instance remains stable but is maybe associated with negative mechanical properties [26, 27]. Unfortunately, the measurements recorded by Markovic et al. [24] evaluating endo-sinus bone volume from pre-operative and post-operative CBCTs are hard to compare with those obtained in our study (two-dimensional). The effect of ongoing apical bone gain has already been reported in a short-term evaluation after 6 months and 2 years [16].

The study outcome does not support the early postulation that the internal / transcrestal approach should be solely reserved to cases where residual bone heights are above 6 mm [5]. Nonetheless, it should be kept in mind that residual bone height should not be too small leading to absence of required primary stability. However, it is not possible to figure out a certain residual height as primary stability depends e.g. on bone quality and implant parameters.

The number of membrane perforations occurring during ISFE (21.7%) was high compared to a previous review (frequency between 3 and 19%) [18]. Only two studies reported higher values of 37.5 and 40.7% [28, 29] whereas many studies did not report the number of perforations. In our study, no effect of membrane perforation on apical bone gain was observed. Comparison of perforated and non-perforated membranes revealed similar longitudinal patterns of apical bone gain. The need for the repair of the Schneiderian membrane demanded by Shlomi et al. [30] should be questioned if graft-free ISFE is used as dislocation of non-endogenous particles into the sinus is excluded. In addition, implant survival of those in perforated sites is quite high [31]. However, epitaxis and thickening of the Schneiderian membrane (as a sign of sinusitis) are the most frequently observed complications following implant penetrations into the sinus [31]. Thus, of course, membrane perforations should be avoided in order to save patients an additional burden and a probably less predictive healing outcome [32]. Regarding the recent study it might be also possible that a greater sample size would have proved a significant difference in bone gain between implants in sites with perforated and non-perforated membranes, respectively. However, this research should not suggest the use of preferably long implants possibly including a higher risk for membrane perforations during implant placement; the message should rather be that placement of an implant of normal length can even be realized using ISFE in smaller residual bone heights.

Mean marginal bone loss of 0.8 mm (distal) and 0.86 mm (mesial) at T1 was within the range reported in a recent review on graft-free sinus-floor elevation [18]. The authors calculated a weighted mean of 0.91 mm for marginal bone loss based on 22 clinical studies with different observation periods combined with a significant effect of the observation period. This is in agreement with our study, in which an increase of mean marginal bone loss to 1.4 mm was found after 5–8 years.

Marginal bone loss was significantly associated with greater initial marginal bone heights. Higher values for initial marginal bone level (distance between first thread and marginal bone level) were caused by deeper insertion of the implants. Between 15 and 50% of longitudinal marginal bone loss could be explained by this variable. On the basis of our data, deeper insertion of the implant, often intended to compensate for the consequences of marginal bone loss, is also a factor promoting marginal bone loss. Therefore, this strategy should be questioned.

Combining apical bone gain and marginal bone loss resulted in a mean overall vertical bone gain of more than 2 mm after 5–8 years, because mean apical bone gain was almost three times higher than marginal bone loss. Short implants have been promoted as a less invasive treatment option for the posterior maxilla, because sinus-floor elevation is avoided [33]. High probability of apical bone gain combined with minimum morbidity of ISFE reduces the attractiveness of short implants placed in the posterior maxilla. Furthermore, minimum marginal bone loss may become significant for short implants.

### Methodical considerations

With regard to implant surgery it is also worth discussing the ISFE approach performed in this study. Summers firstly described the technique as a less invasive method to enable implant placement in the anthropic maxilla. In contrast to the ISFE technique used in our study, Summers exclusively used hand osteotomes to enlarge the recipient site after the sinus floor elevation [4]. The same was done in a study by Caban et al. [34]. Native bone particles may be moved apically which initiate formation of new bone. In the recent study, enlargement of the recipient site was done by consecutive spiral drills and / or hand osteotomes according to the extent of the sinus floor elevation and bone quality. However, it is unclear if native bone particles below the Schneiderian membrane lead to more bone gain as the blood clot itself initiates bone formation. Also contrary to our study, Summers used graft materials which are manipulated under the Schneiderian membrane by use of hand osteotomes [4]. In this study no graft material was used. It should also be acknowledged that no length stoppers for the osteotomes were used in this study. As the ISFE approach is technique sensitive, especially less experienced operators should consider to use.

Another issue which needs discussion is the selection of the bony landmarks. In the recent study the first thread of an implant covered by bone was used as a coronal reference and the bone line directed to the sinus floor (not the sinus floor itself, only the bone level) as apical reference. In contrast to that references, especially if studies target the development of soft tissues, frequently the interproximal height of bone as described by Tarnow et al. [35] is determined and used as reference. However, this was not done in the recent study. Apical bone gain and marginal bone loss were separately calculated for each single implant.

### Strengths and weaknesses

As far as the authors are aware, this study delivers the first evaluation of apical bone gain and marginal bone loss of implants placed in combination with transcrestal / internal sinus-floor elevation without graft in the long-term. However, it should be kept in mind that planning of implant surgery and evaluation of bone heights were based on two-dimensional radiographs. A 3D evaluation (e.g. cone-beam computer tomography, CBCT) would have been yielded advanced information on the surgical sites including bone characteristics and volumetric measurements. Nonetheless, three things regarding this issue should be considered: first, in clinical routine a CBCT is not done in each patient case, second, CBCT comes along with a substantially higher exposure dose which is hard to justify to the patients on a regular basis, especially in a long-term study. Last, the radiographs in this study were taken in parallel technique based on a reference (metal pins in the surgical splint) was performed in the radiograph viewing software which offered length correction. To this end, high inter-rater reliability of linear measurements was confirmed by repeated measurements of 40 radiographs by two independent investigators.

Another issue needing discussion is that the type of suprastructures anchored by the implants was not recorded. Whilst the type of prosthetic restorations has a small but significant impact on clinical success of implants [36] it is also likely that it affects bone gain and loss, respectively. Thus, a possible influence cannot be excluded. The same is true for i.e. the type of cement used. However, the greatest bone gain was seen at the first follow-up visit (before prosthetic management) with decelerated further bone gain, respectively, thereafter.

Clinical parameters such as periodontal measures or lifestyle (i.e. plaque index, gingiva index, smoking behavior) can also affect bone formation. However, in the long-term those parameters hardly differ and those patient-related conditions are therefore difficult to statistically address.

To this end, specific attention should be paid on mobile prostheses during the (initial) healing period. Implant types with a strictly parallel design including the platform are prone to be dislocated into the sinus with the respective consequences if the insertion torque was low (i.e. 5 Ncm). In each case, prostheses have to be grinded and patients should be informed to eat soft food and / or to forgo the prostheses until suture removal.

## Conclusions

Considering the limitations of this clinical study leads to subsequent conclusions:

- Substantial initial bone gain within the first year and decelerated but ongoing apical bone gain afterwards can be expected if implants are placed in combination with ISFE without graft.
- Small residual bone height (coronal bone edge to sinus floor) is associated with a higher likelihood of greater bone gain.
- ISFE without graft can be combined with residual bone height of less than 5 mm if primary stability of the implant has been achieved.
- Further longitudinal studies using three-dimensional measurements based on CBCTs are desirable.

## Data Availability

The datasets used and analyzed in this study are available on reasonable request from the corresponding author (andreas.zenthoefer@med.uni-heidelberg.de).

## Abbreviations

CBCT: Cone-beam computer tomography
mm: Millimeter
n: Case numbers
p: *p* value
SD: Standard deviation
T: Measurement time
ISFE: Internal sinus-floor elevation
